# 

**Published:** 1891-04

**Authors:** 


					﻿JLFR.II-. 1891
OLD SERIES
Vol. xxv
NEW SERieS
Vol. Vlll.-N». 2.
& 1855 O


JOURNAL



H. V. M. MILLER, M. D., LL.D.
VIRGIL 0. HARDON, M. D.
F. W. McRAE, M. D.
L.	P. KENNEDY, A. M., M. D.
M.	B. HUTCHINS, M. D.
L. B. GRANDY, M. D.
<i|RUSHEDBY JAMES RHARRISON&Cd
_________________sBLtlanta.ga. Ji
SUBSCRIPTION I <2.60 A YKAR»
Entered at the Post-Office at Atlanta, Ga., as second-class matter.
				

